# A comparison of peripheral blood stem cell collection outcomes for multiple myeloma; mobilization matters in the era of IMiD induction

**DOI:** 10.1002/jha2.702

**Published:** 2023-05-08

**Authors:** Thea Chandler, Christopher Parrish, Marina Karakantza, Jonathan Carmichael, David Pawson, Gordon Cook, Frances Seymour

**Affiliations:** ^1^ St James's Institute of Oncology Leeds Teaching Hospitals NHS Trust Leeds UK; ^2^ NHS Blood and Transplant Barnsley UK

**Keywords:** myeloma, stem cell mobilization/homing, stem cell transplantation

## Abstract

Collection of peripheral blood stem cells (PBSCs) for autologous stem cell transplant (ASCT) requires mobilization from the bone marrow. There is variation in mobilization choice; during the COVID‐19 pandemic BSBMT&CT guidelines recommended using granulocyte‐colony stimulating factor (G‐CSF) alone to minimize the use of chemotherapy. We report on the impact of mobilization regimen on stem cell collection, and whether IMiD‐containing induction therapy impacts on mobilization and consequently transplant engraftment times for 83 patients undergoing ASCT at Leeds Teaching Hospitals.

Cyclophosphamide plus G‐CSF (cyclo‐G) mobilization yielded more CD34^+^ cells (8.94 vs. 4.88 ×10^6^/kg, *p* = < 0.0001) over fewer days (1.6 vs. 2.4 days, *p* = 0.007), and required fewer doses of salvage Plerixafor than G‐CSF only (13.6% vs. 35%, *p* = 0.0407). IMiD‐containing induction impaired all of these factors. CD34^+^ doses > 8×10^6^/kg were more frequent with Cyclo‐G (62% vs. 11%, *p* = 0.0001), including for those receiving IMiD 1st line induction (50% vs. 13.3%, p = 0.0381). Note that 92.6% of those receiving IMiD‐free inductions were mobilized with Cyclo‐G.

The novel agents used in modern induction regimens (e.g Daratumumab) have been shown to impair yields, increasing the importance of optimizing mobilization regimens in the first instance. Furthermore, as cellular therapies become established in the management of multiple myeloma emerging data highlights the potential benefits of stem cell top up in the management of the haematological toxicities of these therapies. Our findings support re‐adoption of Cyclo‐G as the gold standard for mobilization to optimize PBSC harvesting and ensure sufficient cells for subsequent ASCTs.

## 1 INTRODUCTION

Autologous stem cell transplant (ASCT) has long been the gold standard treatment for eligible patients with newly diagnosed multiple myeloma. ASCT requires prior collection of haematopoetic progenitor cells, almost always by mobilization from the bone marrow into the peripheral blood and subsequent apheresis. This can be achieved by administration of granulocyte‐colony stimulating factor (G‐CSF) either alone or in combination with chemotherapy. The regimens employed for this vary, due to an absence of clear data indicating superiority of one combination over any others. In the UK, no national guidelines have historically been in place and the choice of mobilization agent has been at the discretion of treating centres. In the UK in 2020, patients undergoing ASCT for a malignant condition were mobilized using G‐CSF alone (51%), cyclophosphamide and G‐CSF (Cyclo‐G) (21%), or a combination of G‐CSF and an alternative chemotherapy agent (28%) [1].

Chemo‐mobilization is not without risk, and in response to the COVID‐19 pandemic, to minimize chemotherapy‐associated COVID‐19 mortality and morbidity, the British Society of Blood and Marrow Transplantation and Cellular Therapy (BSBMT&CT) issued a national recommendation for G‐CSF only mobilization [2]. This recommendation resulted in a change in practice in those centres that had historically used Cyclo‐G for mobilization and presents the opportunity for comparison of mobilization outcomes between regimens.

Peripheral blood stem cell (PBSC) yield has been shown to be impacted by factors including mobilization regimen [3, 4] and induction therapy [5, 6, 7]. The treatment landscape for multiple myeloma is constantly evolving, with an increasing emphasis on incorporation of additional therapies into front‐line treatment, followed by prolonged maintenance regimens. For example, the recent Cassiopeia study compared bortezomib‐thalidomide‐dexamethasone (VTD) induction against VTD plus daratumumab, showing a clear improvement in the primary endpoint of stringent complete response after completion of consolidation therapy (29% vs. 20%, *p* = 0.001) [5]. These data have led to widespread adoption of Dara‐VTD as an induction protocol, including recommendation by the National Institute for Health and Care Excellence (NICE) in the UK. Post‐transplant maintenance strategies, typically incorporating immunomodulatory drugs (IMiDs) such as lenalidomide also improved outcomes [8], but use of these drugs has been shown to negatively impact on mobilization yield [6, 9]. Since second transplantation has been shown to be an effective strategy for relapsed myeloma [10], it is necessary to re‐evaluate the optimum mobilization agents in order to ensure adequate stem cell harvesting is achieved at first line to allow subsequent ASCT if later required.

The aim of this retrospective review is to evaluate the impact of mobilization regimen on stem cell collection, and to determine whether IMiD‐containing induction therapy impacts on mobilization and consequently transplant engraftment times.

## 2 METHODS

This was a single centre retrospective study of multiple myeloma patients undergoing ASCT at Leeds Teaching Hospitals NHS Trust, Leeds, UK between 1st January 2016 and 30th September 2021. Eligible patients were those with a confirmed diagnosis of multiple myeloma according to IMWG criteria [11], aged over the age of 18, who had undergone one or two lines of induction chemotherapy and had proceeded to stem cell mobilization. Patients were classified as having received G‐CSF only priming (10mcg/kg for 5 days) or Cyclo‐G (cyclophosphamide 2 g/m^2^ then G‐CSF 5mcg/kg for 10 days). Patients receiving alternative mobilization chemotherapy regimens were excluded. Induction regimens were classified as IMiD‐containing (either thalidomide or lenalidomide) or non‐IMiD‐containing.

Plerixafor was used in accordance with NICE clinical commissioning policy [12] defined as ‘rescue’ treatment given as a second mobilization regimen after an earlier unsuccessful harvest, or ‘pre‐emptive’ treatment given in those with low levels of circulating PBSCs on day of harvest to attempt to prevent the need for a second mobilization. Stem cell targets were predetermined by treating physicians and the decision to attempt further days of apheresis made clinically in accordance with collection reports on the day.

While a minimum cell dose of 2 × 10^6^ CD34^+^/kg per ASCT is required for safe engraftment, higher doses of 3–5 × 10^6^/kg are associated with optimal engraftment [13]. It is also common practice to collect a sufficient stem cell dose for two ASCTs to enable future salvage transplant if required. Therefore, our CD34^+^ minimum target was >4 × 10^6^/kg, with an optimal target of >8 × 10^6^/kg.

Mobilization outcomes included CD34^+^ yield, duration of harvest, rescue Plerixafor use and infective complications. Engraftment was defined as the first of three consecutive days of achieving a sustained peripheral blood neutrophil count of >0.5×10^9^/L.  Mobilization data were included in the analysis regardless of whether the patient progressed to transplant. Groups were compared using the Mann–Whitney or chi‐squared tests.

## 3 RESULTS

Eighty‐three patients with multiple myeloma underwent 86 mobilization procedures between January 2016 and September 2021. One patient was mobilized four times within this period for first line and salvage transplants; all other patients underwent a single mobilization attempt. Baseline characteristics are summarized in Table 1. Sixty‐six harvests used Cyclo‐G and 20 used G‐CSF.

**TABLE 1:** Baseline characteristics of myeloma patients undergoing mobilization for autologous stem cell transplant (ASCT).

	**Cyclo‐G** (*n* = 66)	**G‐CSF** (*n* = 20)	
**Age** (years)	60	61.5	*p* = 0.571
**Sex**			
Male/Female (%)	56/44	70/30	*p* = 0.287
**1st line of therapy** (%)	89	90	*p* = 0.938
**IMiD‐containing 1st line therapy** (%)	20.3	83.3	*p* = < 0.0001
**Cycles of induction pre‐apheresis** (number)	5.3 (4–14)	4.1 (0–6)	*p* = 0.0021
**Time from apheresis day 1 to transplant day 0** (days)	60 (26–247)	82 (35–143)	

### 3.1 Apheresis yields

**FIGURE 1:**
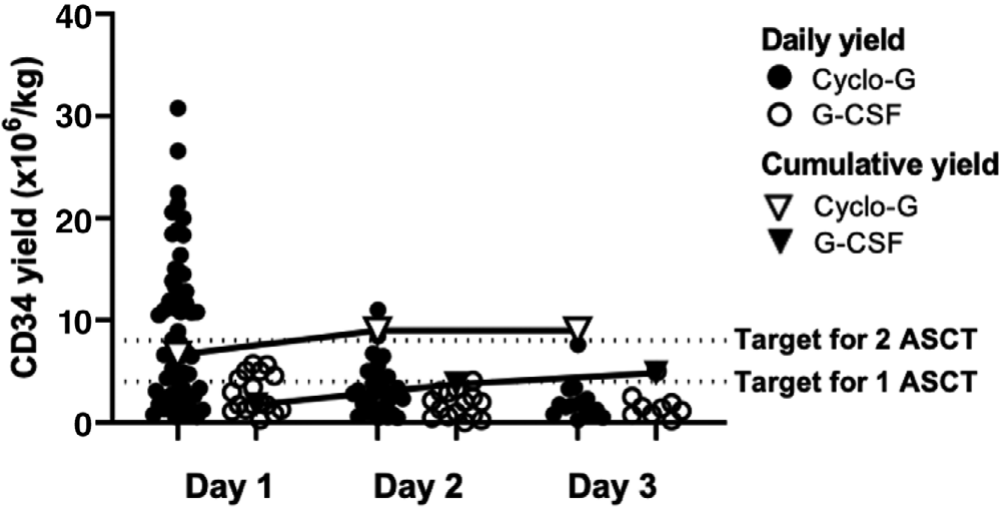
Comparison of daily CD34^+^ yields for the first three days of apheresis, based on mobilization regimen.

Eighty‐six per cent of harvests achieved the minimum target, with more failed harvest being seen after G‐CSF only mobilization (10.6% vs. 25% *p* = 0.1) (Table 2). The improved mobilization of PBSCs with Cyclo‐G is reflected in increased pre‐apheresis day 1 CD34^+^ counts (95 vs. 46.94 × 10^6^/kg, *p* = 0.06). Mobilization with Cyclo‐G yielded higher CD34^+^ doses (8.94 vs. 4.88 × 10^6^/kg, *p* < 0.0001) and required fewer apheresis days (1.6 vs. 2.4 days, *p* = 0.007) (Figure 1). Optimal harvest yields of >8 × 10^6^/kg were more frequently attained with Cyclo‐G mobilization (62% vs. 11%, *p* = 0.0001) (Figure 1), including for those receiving IMiD‐containing 1st line induction (50% vs. 13.3%, *p* = 0.0381).

**TABLE 2:** Comparison of apheresis outcomes, based on mobilization regimen and IMiD induction.

	**Cyclo‐G** (*n* = 66)	**G‐CSF** (*n* = 20)	
**Mean CD34^+^ dose** (×10^6^/kg)	8.94	4.88	*p* = <0.0001
**Days of collection** (number)	1.67 (1–4)	2.4 (1–5)	*p* = 0.007
**Plerixafor use** (%)	13.6	35	*p* = 0.0315
**Failed harvest** (%)	10.6	25	*p* = 0.1
**1st line patients achieving** **CD34^+^ dose > 4 × 10^6^/kg** (%)	97	83	*p* = 0.045
**1st line patients acheiving** **CD34^+^ dose > 8 × 10^6^/kg** (%)	62	11	*p* = <0.0001
**1st line IMiD patients achieving** **CD34^+^ dose > 4 × 10^6^/kg** (%)	100	86.7	*p* = 0.1887
**1st line IMiD patients achieving** **CD34^+^ dose > 8 × 10^6^/kg** (%)	50	13.3	*p* = 0.0381

### 3.2 Impact of IMiD‐containing induction

**FIGURE 2:**
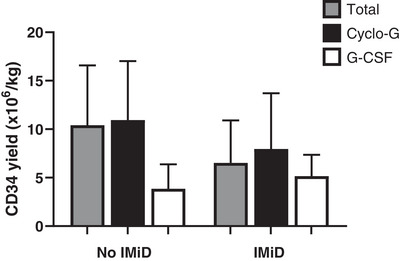
Mean CD34^+^ yields by induction type and mobilization regimen.

CD34^+^ yields across the entire cohort were lower after IMiD‐containing induction (5.18 vs. 8.98 × 10^6^/kg, *p* = 0.00003, *n* = 32) although there was a trend towards higher yields when Cyclo‐G mobilization was used (5.8 vs. 4.8 × 10^6^/kg, *p* = 0.34) (Figure 2). In patients mobilizing after 1st line IMiD therapy (n = 27), Cyclo‐G mobilization resulted in higher yields (8.51 vs. 5.18 × 10^6^/kg, *p* = 0.0321). Note that 34.4% of patients mobilizing after IMiD‐containing therapy required Plerixafor, as opposed to 9.3% of IMiD‐free inductions (*p* = 0.03). IMiD‐containing induction resulted in a greater apheresis requirement (median 2 days vs. 1 day, *p* = < 0.0001). The mean number of cycles of induction was similar (5.3 [3‐14] vs. 4.1 [0–6], Cyclo‐G vs. G‐CSF *p* = 0.021). There was no significant correlation between number of induction cycles and CD34^+^ yield or days of apheresis in either groups. (Median CD34^+^ dose; 6.67 vs. 7.26, ≤ four vs. > four cycles, *p* = 0.8537, median of 2 days apheresis in both groups).

### 3.3 Impact on Plerixafor use

**FIGURE 3:**
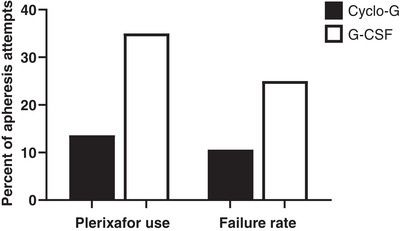
Rescue plerixafor use and failure rates by mobilization regimen.

The use of Plerixafor in this population was ‘pre‐emptive’, except for one patient mobilized with G‐CSF who previously failed an attempt with Cyclo‐G and was given ‘rescue’ Plerixafor. More patients mobilized with G‐CSF required Plerixafor (35% vs. 13.6%, *p* = 0.0407) (Figure 3). Of the nine Cyclo‐G mobilizations that required Plerixafor, one patient received three doses, two received two doses and the remaining received one dose. Of the seven G‐CSF mobilizations that required Plerixafor, two received two doses and the remaining one dose. Patients receiving mobilization with G‐CSF + Plerixafor were less likely to progress to transplant than Cyclo‐G + Plerixafor (53% vs. 88% *p* = 0.1523).

### 3.4 Mobilization complications

Five patients receiving Cyclo‐G were hospitalized, including one with neutropenic sepsis. All five patients recovered and progressed to successful transplant. There were no recorded infective complications prior to transplant from mobilization with G‐CSF.

### 3.5 Engraftment outcomes

Seventy‐four transplants were included in outcomes analysis, 12 were excluded for either failure to progress to transplant (*n* = 10) or patient death prior to day 100 (*n* = 2). Mobilization regimen did not impact the time to absolute neutrophil engraftment (17.02 vs. 17.5 days, Cyclo‐G vs. G‐CSF, *p* = 0.9307). This was also true for transplants following IMiD‐containing induction (*n* = 24) (16.69 vs. 17.36 Cyclo‐G vs. G‐CSF, *p* = 0.8968).

## 4 DISCUSSION

Determination of the optimal strategy for maximizing stem cell harvest yields is highly advantageous, both to ensure the highest possible proportion of suitable patients can proceed to one or more autologous transplants, and to minimize the patient and healthcare burden of repeated harvesting attempts. It is widely accepted that higher CD34^+^ doses ensure transplant safety and shorten haematopoetic reconstitution [13]; however, the underpinning data largely predate the adoption of novel therapies, which may alter the engraftment kinetics and autograft cellular constituents.

Our findings highlight important considerations for the present‐day clinician preparing myeloma patients for autologous transplant. Mobilization with Cyclo‐G resulted in higher yields, over a shorter time frame and required fewer doses of rescue Plerixafor. The differences in stem cell yields are reflected in the UK national data for autologous mobilizations in 2020 (median yield: Cyclo‐G vs. G‐CSF 7.5 vs. 4.9 × 10^6^/kg) [1]. The addition of IMiD‐containing induction impaired stem cell yields and resulted in higher percentage of patients requiring Plerixafor, returning for consecutive days of apheresis.

35% of G‐CSF only mobilizations in this sample required Plerixafor, which although high is considerably lower than the 60% seen for autologous transplants nationally [1]. It is difficult to compare the use of Plerixafor in our population to that of other centres worldwide. The percentage of patients on G‐CSF only mobilization requiring rescue Plerixafor is probably higher in Europe and United States where the threshold for use is <10×10^9^/L [14]. In our centre, Plerixafor use is according to NICE guidelines, which recommend a rescue regimen for poor mobilizers in whom the CD34^+^ cell count is <15×10^9^/L on the day of predicted day of stem cell harvest [12].

In the UK, where commissioning arrangements for Plerixafor mean that repeated use is not available, avoiding rescue doses in early harvest attempts means an additional mobilization option for patients who later elect to undergo subsequent or tandem transplants. With the NHS indicative price of Plerixafor equating to £4882.77 per 20 mg vial (the standard dose for patients up to 84 kg), there are potential cost savings, especially when combined with the reduced requirements on apheresis units. Financial implications are beyond the scope of this research; however, Lazzaro et al. developed a decision tree‐supported cost‐effectiveness analysis model for myeloma patients eligible for autograft. They concluded that, in Italy, G‐CSF alone (± on‐demand Plerixafor) was more cost effective than cyclophosphamide + G‐CSF (± on‐demand Plerixafor), €8039.85 and €9238.44 per patient, respectively [15].

Additionally, patients receiving mobilization with G‐CSF + Plerixafor were less likely to progress to transplant than Cyclo‐G + Plerixafor (53% vs. 88% *p* = 0.1523). When analysing the reason these patients were not transplanted, 50% of patients receiving G‐CSF + Plerixafor showed signs of disease relapse between mobilization and planned transplant date (*n* = 2). One patient failed mobilization and one patient elected not to proceed. All patients who did not progress to transplant after Cyclo‐G + Plerixafor had failed to mobilize sufficient PBSCs. Only one patient mobilized with Cyclo‐G showed signs of disease relapse and did not progress.

There was no significant difference detected in time to neutrophil engraftment, showing cyclophosphamide priming did not impair time to neutrophil recovery. The negligible impact of Cyclo‐G versus G‐CSF mobilization on subsequent neutrophil engraftment has been previously reported in the literature [16]. The impact of Plerixafor on transplant outcomes is difficult to evaluate, due to the confounding factor that those receiving Plerixafor are largely poor mobilizers and therefore more likely to have poorer OS and PFS [17]. In studies selecting for predicted or proven poor mobilizers, Plerixafor use has been shown not to affect transplant outcomes [17, 18] suggesting there is not an advantage to using G‐CSF + Plerixafor over Cyclo‐G. Conversely, mobilization using cyclophosphamide incurs risk associated with the use of alkylating agents; namely febrile neutropenia and increased episodes of hospitalisation. Our data report higher rates of hospitalisation within the Cyclo‐G group, but that there was no subsequent impact on neutrophil engraftment or success of transplant in these patients.

Limitations of this retrospective review include small sample size and, notably, that 92.6% of those receiving non‐IMiD‐containing inductions were mobilized with Cyclo‐G, meaning differences may be attributable not only to mobilization regimen but also in part to induction therapy. This confounding factor results from the evolutionary timeline of myeloma therapies; in our centre, mobilizations with G‐CSF only took place during the early years of the COVID‐19 pandemic, where IMiD‐based induction formed the vast majority of first line treatments. In addition, the follow‐up of our study is too short for evaluation of progression‐free survival to be meaningful at this point and is likely to be confounded by the variations in induction regimens.

In the era of IMiD‐based induction and continuous IMiD maintenance, our data highlight the negative impact of IMiD treatment on CD34^+^ mobilization and the importance of optimizing stem cell collection in the first line setting. Highly impressive results from recent studies [5, 7, 19] mean that daratumumab has been adopted as a component of front line therapy in multiple healthcare settings, and this has been shown to further impair stem cell yields, increasing the importance of optimizing mobilization regimens. In addition, the treatment landscape in myeloma is rapidly adopting novel cellular therapies in the form ofchimeric antigen receptor (CAR)‐T cells and T cell engager antibodies. These highly effective treatments are associated with significant and persistent haematological toxicity. Rejeski et al. have recently developed the predictive CAR‐Haematotox score, which identifies patients at high risk of developing significant post CAR‐T bone marrow aplasia and infections [20]. In the setting of myeloma 45% of patients have a high risk score and 30% of these patients develop post infusion aplasia [21]. Autologous stem cell boost has been shown to result in rapid and sustained haematological recovery with improved outcomes in patients with post CAR‐T neutropenia [22]. The availability of cryopreserved autologous PBSCs in myeloma may therefore have an important role beyond the traditional ASCT. Accordingly, we would advocate that Cyclo‐G be considered the standard of care in order to achieve sufficient PBSC yields to enable subsequent ASCTs or autologous stem cell boosts, especially in those receiving IMiD‐containing induction therapy.

## AUTHOR CONTRIBUTIONS

TC designed the research, collected and analysed data and wrote the manuscript. FS and CP designed the research, analysed data and edited the manuscript. FS performed statistical analysis. MK and DP contributed data from NHSBT. All authors provided feedback on the manuscript.

## CONFLICT OF INTEREST STATEMENT

The authors have no competing interests to declare

## FUNDING INFORMATION

This work did not receive any specific grant from funding agencies in the public, commercial or not‐for‐profit sectors.

## ETHICS STATEMENT

None

## Data Availability

The datasets generated during and/or analysed during the current study are available from the corresponding author upon reasonable request.
